# Investigation of Electroplastic Effect on Four Grades of Duplex Stainless Steels

**DOI:** 10.3390/ma12121911

**Published:** 2019-06-13

**Authors:** Claudio Gennari, Luca Pezzato, Enrico Simonetto, Renato Gobbo, Michele Forzan, Irene Calliari

**Affiliations:** 1 Department of Industrial Engineering, University of Padua Via Marzolo 9, 35131 Padova PD, Italy; luca.pezzato@unipd.it (L.P.); irene.calliari@unipd.it (I.C.); 2 Department of Industrial Engineering, University of Padua Via Venezia 1, 35131 Padova PD, Italy; enrico.simonetto.1@unipd.it; 3 Department of Industrial Engineering, University of Padua Via Gradenigo 6/A, 35131 Padova PD, Italy; renato.gobbo@unipd.it (R.G.); michele.forzan@unipd.it (M.F.)

**Keywords:** duplex stainless steels, electroplastic effect, tensile test, stacking fault energy, electrically assisted forming, electron stagnation theory, current distribution, mechanical properties, uniform elongation

## Abstract

Since the late 1950s, an effect of electrical current in addition to joule heating on the deformation of metals called the Electroplastic Effect (EPE) has been known. It is used nowadays in the so-called Electrically Assisted Forming (EAF) processes, but the understanding of the phenomenon is not very clear yet. It has been found that EPE increases the formability of high stacking fault energy (SFE) materials, while low SFE materials reach fracture prematurely. Since Duplex Stainless Steels (DSSs) possess a microstructure consisting of two phases with very different SFE (low SFE austenite and high SFE ferrite) and they are widely used in industry, we investigated EPE on those alloys. Tensile tests at 5 A/mm^2^, 10 A/mm^2^ and 15 A/mm^2^ current densities along with thermal counterparts were conducted on UNS S32101, UNS S32205, UNS S32304 and UNS S32750. The DSS grades were characterized by means of optical microscopy, X-ray diffraction and their mechanical properties (ultimate tensile strength, total elongation, uniform elongation and yield stress). An increase in uniform elongation for the electrical tests compared to the thermal counterparts as well as an increase in total elongation was found. No differences were observed on the yield stress and on the ultimate tensile strength. Un uneven distribution of the current because of the different resistivity and work hardening of the two phases has been hypothesized as the explanation for the positive effect of EPE.

## 1. Introduction

In 1959, Machlin [1] observed an increase in elongation and a reduction of the yield stress and the flow stress of a single crystal of sodium chloride when deformed under an applied voltage. In the subsequent years, two main groups started to investigate the effect of electrical current on the deformation of various metallic and non-metallic materials. Pioneering researches were conducted in the USA by Conrad and his team [2,3,4,5,6,7] and in the Soviet Union by Troitskii and his people [8,9,10,11,12,13], who concluded that the enhancement of the formability and the reduction of the forming forces cannot be ascribed solely to the joule heating but also to an a-thermal effect, which they called the Electroplastic Effect (EPE). The discovery of such effect has led to a new way of forming metallic materials called Electrically Assisted Manufacturing (EAM), which is applied to a variety of processes such as rolling [14,15], drawing [8,16], cutting [16,17,18], forging [19,20], sintering [21,22], and bending [23,24], among others.

Many theories have been formulated to explain the EPE such as the electron wind force by Kravchenko [25], who supposed that drifting electrons can exert a force on moving dislocations and ease their motion, which was later confirmed by experiments done by Bolko et al. [26] and Conrad and his team [6,27]. Other researchers claimed that the electron wind force is negligible and the EPE is due to depinning of dislocations from weak obstacles thanks to the induced magnetic field, which shifts the state of a dislocation’s core from singlet to triplet [28,29]. Fan et al. observed that pulses of high current density induced grain boundary cavitation in 70/30 brass under uniaxial tensile test [30]. Magargee demonstrated that each material has a different current density sensitivity for EPE to occur, which depends on the resistivity of the material (the higher the resistivity, the lower the threshold) [31]. A dependence from the Stacking Fault Energy (SFE) on the occurrence of the EPE has also been observed [32]. An interesting theory was proposed by Ruszkiewicz et al. [33]: stagnation of electrons in the proximity of obstacles increases the electron to atom ratio, decreasing the bond energy and eventually easing the breaking and reforming of metallic bonds favoring plastic deformation. Many experiments have been done concerning the EPE on different metallic materials such as aluminum [34,35,36], magnesium [15,37,38,39], and austenitic stainless steels [40,41,42]; some biphasic materials such as titanium [43], a dual phase steel [44], and brass [30]; only one paper has been found on Duplex Stainless Steels (DSSs) regarding the change in texture after electropulsing treatment at room temperature [45].

DSSs are a peculiar type of stainless steels in which the austenitic and the ferritic phase are both present. They are widely used in a variety of industrial applications, thanks to their high corrosion resistance and high mechanical properties compared to the austenitic stainless steels [46,47,48]. The metallurgy of DSSs is quite complicated because the thermal history can affect their mechanical properties, since they suffer from secondary phases precipitation in a wide range of temperatures (450–1000 °C). The most observed and detrimental secondary phases are chi-phase, sigma-phase, chromium nitrides and carbides followed by L-phase, R-phase, pi-phase and Laves phase, the spinodal decomposition of ferrite and the eutectoidic decomposition of ferrite into secondary austenite and chromium nitrides. Because of the sensitivity to secondary phases precipitation and the needs of a balanced microstructure, DSSs have to undergo a solubilizing heat treatment at a temperature that depends on the DSS’s grade to solubilize the secondary phases and to obtain an equal amount of austenite and ferrite. DSSs solidify in a fully ferritic microstructure, at a temperature between 1400 °C and 1500 °C (depending on the DSS grade) and, as the alloy cools down, austenite starts to precipitate from the ferrite matrix with a defined orientation relationship with respect to ferrite such as Kurdjumov–Sachs (K–S) or Nishiyama–Wasserman (N–W) or as incoherent phase boundary [49,50]. Solubilizing heat treatment must be conducted at temperature higher than 1030 °C (depending on the alloy composition) to dissolve the unwanted secondary phases and to obtain an equal volume fraction of austenite and ferrite. The cooling phase is critical and must be conducted at a high cooling rate to freeze the microstructure to room temperature and limit the time the alloy spends in the critical temperature range, in which secondary phases precipitation occurs. Hence, the thickness of the components is a critical parameter because it influences the cooling rate. DSSs must be free of secondary phases because even a small volume fraction can affect the mechanical properties, in particular impact toughness [51,52] and corrosion resistance [53,54]. Austenite and ferrite, obviously, present different crystal structures, the former being face-centered cubic (FCC) and the latter body-centered cubic (BCC). More importantly, they have very different SFE. SFE is an intrinsic property of a crystal structure and it depends on the electron to atom ratio (i.e., the ratio of valence electrons to atoms present in an alloy) and on the temperature [55]. FCC materials with low SFE have a higher probability of dislocations splitting into partial leading to a planar glide during plastic deformation since split dislocations act as an obstacle to the cross-slip mechanism. Conversely, SFE in BCC materials is quite high, which changes the way dislocations move and interact with each other and, moreover, plays an important role at higher temperature since atomic mobility is enhanced and high SFE materials tend to recover rather than recrystallize.

The investigation of the EPE on DSSs is very interesting since they have a high SFE BCC matrix in which is dispersed a low SFE FCC phase. EPE has been observed to influence more BCC materials rather than FCC ones [56], thus the investigation of EPE on these grades of stainless steels could improve the knowledge of this phenomenon. The objective of the study was to investigate the influence of electrical current on the plastic flow behavior of materials that possess two phases with very different SFE and if there is any influence on the insurgence of the EPE with respect to different grades of DSS.

## 2. Materials and Methods

Four grades of DSS provided by the Italian division of Outokumpu S.p.A., namely two lean DSS grades (UNS S32101 and UNS S32304), a standard DSS (UNS S32205) and a super DSS (UNS S32705), were tested. Their compositions are summarized in Table 1. All materials were supplied in the form of warm rolled sheets of 1 mm thickness except UNS S32101, which was 3 mm thick. The specimens for the tensile tests were prepared according to ASTM E8/E8M 16a.

**Figure 1:**
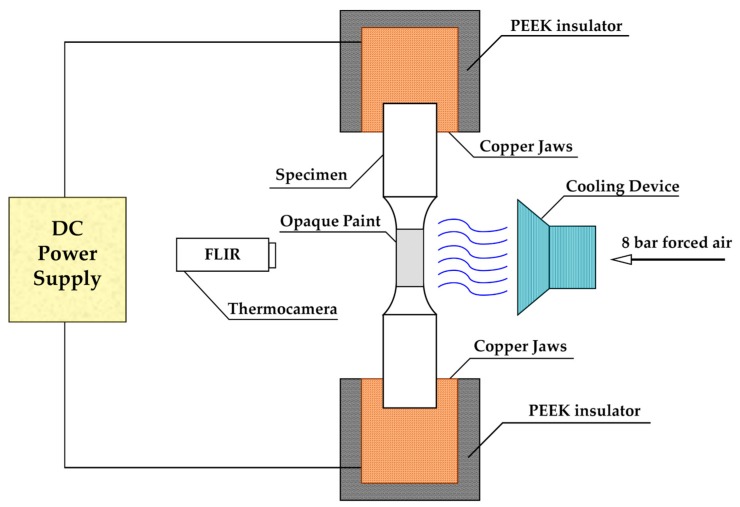
Schematic of tensile test machine setup for electroplastic tensile tests.

Since UNS S32101 suffers from Strain Induced Martensite (SIM) formation [57], all tensile tests were conducted at a strain rate of 10^−1^ s^−1^ to avoid Transformation Induced Plasticity (TRIP) effects. A direct current power supply produced by Powerel s.r.l (Montecchio Maggiore, Vicenza, Italy) is able to deliver a maximum of 6000 A at 10 V. It was coupled with the tensile test machine through self-made copper jaws embedded in PEEK, to electrically isolate the specimen with respect to the frame of the tensile test machine. The tensile tests were conducted on an MTS 322 tensile test machine (MTS Systems Corporation, Eden Prairie, MN, USA) modified as depicted in Figure 1. The tensile test machine was driven by dedicated software. Strain was evaluated by the crosshead movement while stress was collected by a load cell mounted on top of the MTS 322.

All steels were tested with three different continuous current densities (5 A/mm^2^, 10 A/mm^2^ and 15 A/mm^2^). The temperature was recorded for the total duration of the test by means of a FLIR A40 thermal camera (FLIR Systems, Wilsonville, OR, USA). The specimens were coated with a black heat resistant paint to stabilize the emissivity. The thermal tests were conducted at the same temperature and strain rate as the electrical ones with the aid of a self-made heating chamber coupled with the tensile test machine. To reduce joule heating effect, air flow at 8 bar was blown through two nozzles during the electrical tensile tests. Current densities were chosen in order not to overcome 0.5 T_h_, which is the homologous temperature, defined as the ratio between the test temperature and the melting temperature of the alloy. Temperatures higher than 0.5 T_h_ can lead to secondary phase precipitation, enhance the diffusion process in the steel and introduce new dislocation dynamics, which would be difficult to take into account to decouple the effect of electrical current from the raise in temperature. Three tests per test condition were conducted, and the errors are presented as the standard deviation of the data collected for each test.

X-ray diffraction (XRD) measurements were conducted along the rolling direction on the as-received material by mean of a Siemens D500 X-ray diffractometer (Munich, Germany) using Cu Kα radiation with 2θ ranging from 30° to 100° (0.05° step and 5 s counting time per step). Rietveld analyses were conducted by mean of Maud© software (version 2.91, Luca Lutterotti, University of Trento, Trento, Italy) to calculate the volume fraction of the different phases.

Microstructure of the as-received samples was analyzed on a Leica DMRE optical microscope (LEICA Microsystems, Wetzlar, Germany) after grinding up to 1200 grit SiC paper, mirror polishing with polycrystalline diamond suspension (6 µm and 1 µm) and etching with modified Beraha solution.

## 3. Results

### 3.1. Characterization of the As-Received Material

**Figure 2:**
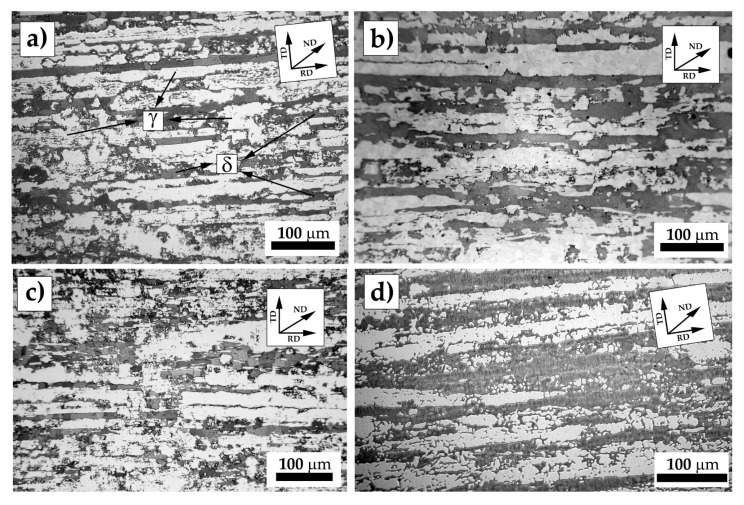
Microstructures of the as-received materials: (**a**) UNS S32101; (**b**) UNS S32205; (**c**) UNS S32304; and (**d**) UNS S32750. Etching solution was modified Beraha. RD, rolling direction; TD, transversal direction; ND, normal direction. Austenite and ferrite are depicted as γ and δ, respectively, in micrograph (**a**).

The as-received materials showed a microstructure composed of austenite grains oriented along the rolling direction dispersed in a ferrite matrix (Figure 2). The rolling process was conducted in the cold/warm regime, as can be seen by the fragmented morphology of the austenite in Figure 2, and the modestly banded ferrite (Figure 2d).

XRD patterns of the as-received materials were acquired to verify the presence of secondary phases and to calculate the volume fraction of the constituents.

**Figure 3:**
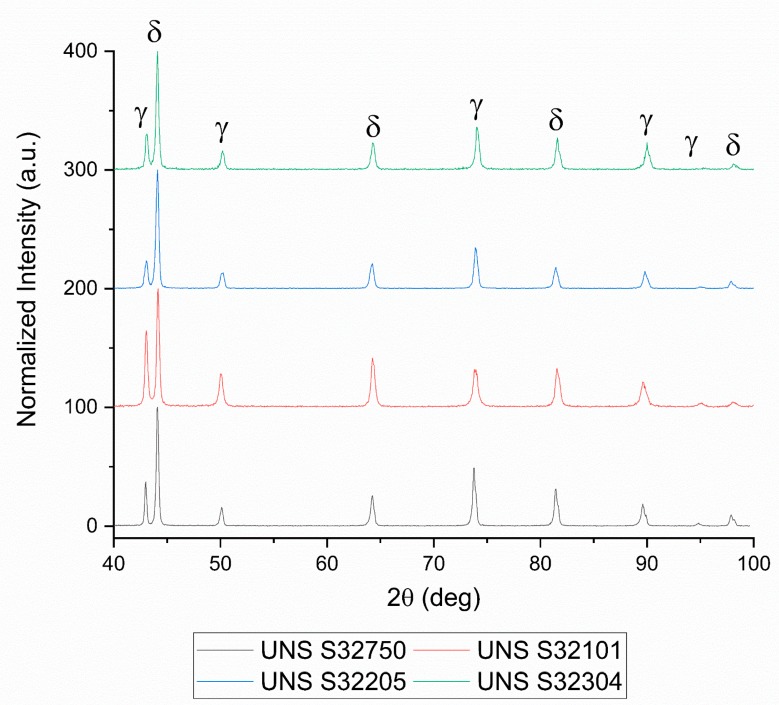
X-ray diffraction patterns of the tested material.

Figure 3 presents the normalized intensity XRD patterns with only the peaks of the two main constituents of DSSs, such as austenite and ferrite, depicted, respectively, as γ and δ. Firstly, secondary phase’s peaks should be visible at lower diffraction angle because of their bigger crystalline cell compared to austenite and ferrite. Secondly, since they grow inside the ferritic phase, the intensity of the ferrite peaks should also be reduced if secondary phases are present. It is therefore clear that the as-received materials were free of secondary phases. Evidence of the rolling process was observed in the XRD pattern: the peaks were much broader than the solution treated sample [57] and the height of the main peaks was different from the theoretical XRD pattern of austenite and ferrite, which translates to a modestly texturized microstructure.

Rietveld analysis on XRD patterns were conducted to calculate the volume fraction of austenite and ferrite. The results are summarized in Table 2. These data confirm the results obtained with image analysis performed on optical microscopy micrographs. All DSS grades showed a well-balanced microstructure with approximately equal volume fraction of austenite and ferrite.

### 3.2. Temperature and Current Regime

The increase in temperature during plastic deformation can be calculated as follows:

(1) $$\Delta T\left({\bar{\varepsilon }}^{p}\right)=\int_{0}^{{\bar{\varepsilon }}_{max}^{p}}\frac{\beta }{\rho C_{p}}\bar{\sigma }\left({\bar{\varepsilon }}^{p}\right)d{\bar{\varepsilon }}^{p},$$

where $\bar{\sigma }\left({\bar{\varepsilon }}^{p}\right)$ is the evolution of stress during plastic deformation, ρ is the density of the material, $C_{p}$ is the specific heat at constant pressure and β is the Quinney–Taylor parameter [58] that describes the fraction of energy that is converted into heat during plastic deformation. The Quinney–Taylor parameter can be considered constant, even though it can vary during plastic strain. In the case that plastic flow is described by a power law, such as Hollomon formulation or Johnson–Cook model, β can be expressed as:

(2) $$\beta \left({\bar{\varepsilon }}^{p}\right)\approx 1-n{\left(\frac{{\bar{\varepsilon }}^{p}}{\varepsilon_{0}}\right)}^{n-1},$$

where *n* is the work hardening exponent of the material and $\varepsilon_{0}$ is the strain at yielding.

The tests were performed at a strain rate of 10^−1^ s^−1^, hence heat exchange with the environment was negligible because of the short duration of the test. Overall, the increase in temperature calculated due to plastic deformation was between 70 °C and 150 °C depending on the fracture strain (Hollomon formulation was used to describe the material behavior), consistent with the thermal camera determinations.

**Figure 4:**
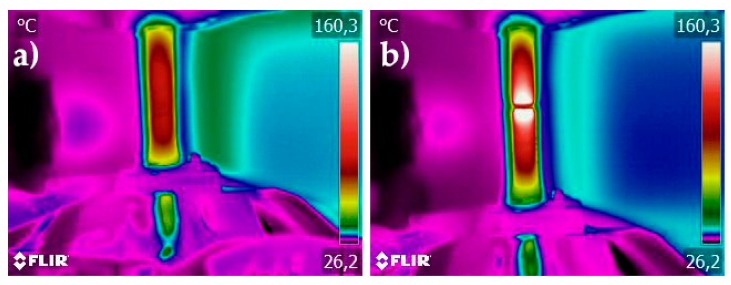
Thermal images of UNS S32101: (**a**) before the tensile test; and (**b**) immediately after fracture. The tensile test was conducted at 5 A/mm^2^.

Figure 4 shows the thermal images of UNS S32101 before the tensile test (Figure 4a) and immediately after fracture (Figure 4b) under 5 A/mm^2^ current density. The temperature were acquired along a line in the middle of the sample. To get a more precise temperature measurement for the lower current density test, the temperature range was limited via software up to 160.3 °C. In Figure 4b, a peak temperature (white region) out of the measurement range corresponding to the fracture surfaces is present because of the local plastic instability, which decreases the cross section, increasing the local current density causing higher joule heating of the region.

**Figure 5:**
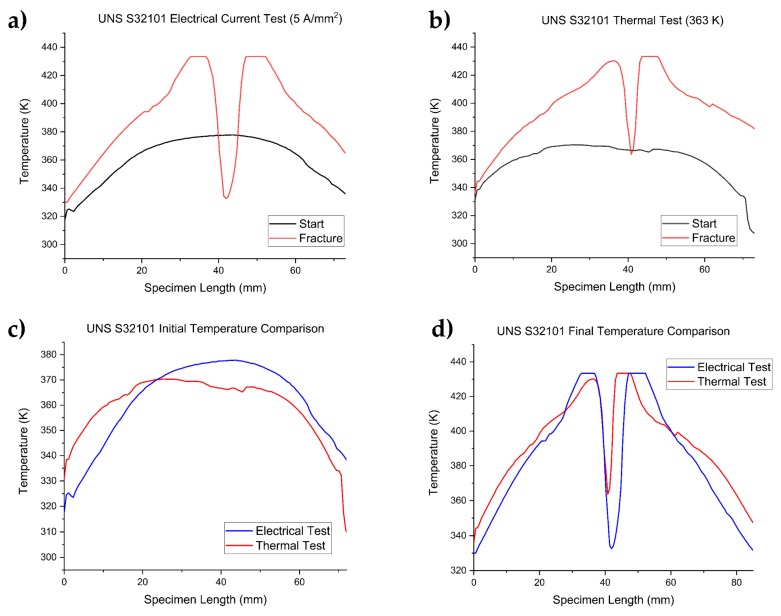
Evolution of temperature along the specimen length of UNS S32101: (**a**) tensile test at 5 A/mm^2^; (**b**) tensile test at 363 K; (**c**) comparison between temperature regimes of specimen tested at 5 A/mm^2^ and the corresponding thermal test at 363 K at the beginning of the tensile test; and (**d**) comparison between temperature regimes of specimen tested at 5 A/mm^2^ and the corresponding thermal test at 363 K right after fracture occurred.

Figure 5 shows the evolution of the temperature along the gauge length of the test conducted at 5 A/mm^2^ and its thermal counterpart at the beginning of the tensile test and after reaching the fracture, in order to compare the imposed thermal regime with the one caused by the electrical current. Only the temperature regime of the test conducted at 5 A/mm^2^ and its thermal counterpart for the UNS S32101 are reported as an example, since all DSSs showed the same trend.

Figure 5a,b shows the evolution of temperature before the test and after the fracture of the tensile test conducted with a current density of 5 A/mm^2^ and the corresponding thermal test. Temperature along the specimen at the beginning of the test was not constant due to the copper jaws that acted as a thermal sink. Nevertheless, the temperature regime imposed in the corresponding thermal test (Figure 5c,d) is in good agreement with the electrical current test.

As stated above, the higher joule heating of the necked region is much more evident in Figure 5d, which compares the temperature regime of the current test at 5 A/mm^2^ and the corresponding thermal test for UNS S32101 after reaching failure. The two peaks in the graph are the temperature of the fracture surfaces: it can be noted that the peaks related to the 5 A/mm^2^ test are clipped because of the software upper limit imposed during the recording of the temperature. For this reason, and because of the increased current density after localized plastic instability, total elongation is not a reliable parameter to consider when analyzing the influence of electrical current during plastic deformation. Nevertheless, some considerations can be made. Thermal regimes of the other DSS grades are not shown here but the same results were obtained.

**Figure 6:**
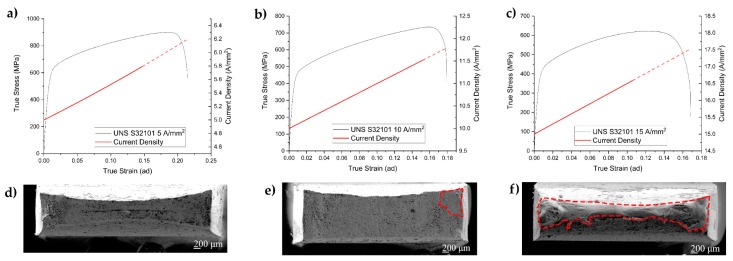
Current density evolution during the tensile test superimposed on the flow stress curve of the UNS S32101 and corresponding fractographies: (**a**,**d**) 5 A/mm^2^; (**b**,**e**) 10 A/mm^2^; and (**c**,**f**) 15 A/mm^2^.

The evolution of the current density during the tensile test was calculated considering the diminishing of the cross section imposing the conservation of the volume. The true stress–strain curves at 5 A/mm^2^, 10 A/mm^2^ and 15 A/mm^2^, together with the evolution of the current density during the test for UNS S32101 DSS grade, are shown in Figure 6a–c as an example. It can be noted that the current density varied during the test from the nominal 5 A/mm^2^ to approximately 5.8 A/mm^2^, from 10 A/mm^2^ to 11.5 A/mm^2^ and from 15 A/mm^2^ to 16.6 A/mm^2^ in correspondence with the maximum uniform elongation. The change from solid to dashed line denotes the insurgence of plastic instability, during which the calculation for the current density evolution did not hold because of necking. After uniform elongation, local plastic instability caused an abrupt increase in current density due to necking, which can cause localized melting of the specimen (area between dashed red line in Figure 6e,f). Some melted regions were observed in the fracture surfaces for the tests conducted at current densities of 10 A/mm^2^ and 15 A/mm^2^, as shown in Figure 6e,f.

The mean temperature reached by the different DSS grades subjected to the current densities used in the tests are summarized in Table 3. Current density and homologous temperature of the investigated samples are also shown in Table 3.

**Figure 7:**
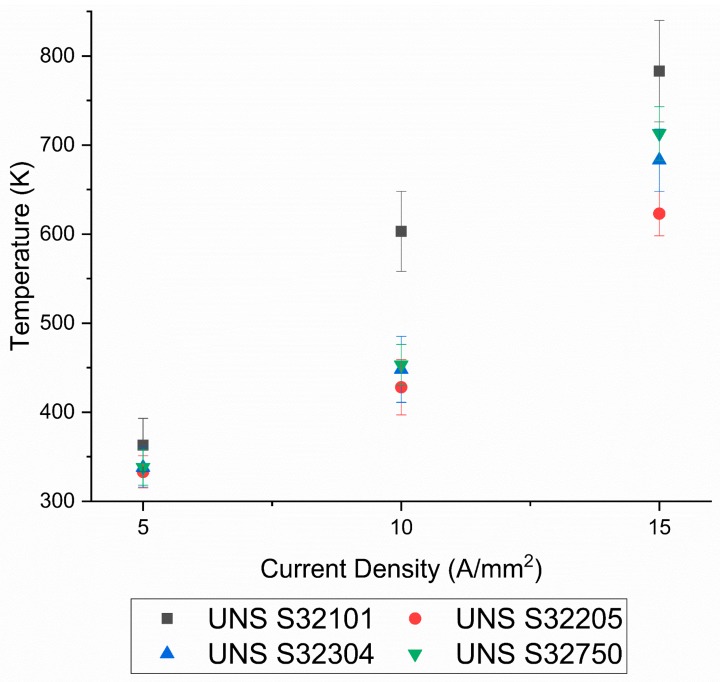
Mean temperatures reached by DSS grades due to the applied current densities.

It was decided to limit the maximum temperature of the tested materials up to 0.5 T_h_ for various reasons: (a) higher temperatures lead to different dislocation dynamics, which are difficult to take into account, and it would be complicated to separate the effect of the electrical current from that of the temperature; (b) the influence of electrical current on secondary phase precipitation and spinodal decomposition of DSSs is not clear and no relevant literature has been found; (c) at higher current densities, the cooling device was not able to efficiently limit joule heating; and (d) the climatic chamber was not able to reach such high temperatures. For more clarity, graphical transposition of Table 3 is shown in Figure 7. UNS S32101 stands out as its increase in temperature was definitely higher than the other DSS grades. The composition of the tested DSS grades did not vary significantly enough to affect the specific heat at constant pressure or the electrical conductivity, hence the higher temperature reached by the UNS S32101 was because of the higher thickness of the sheet. The same geometry for all specimens was used, except for the thickness. UNS S32101 has a lower surface to volume ratio since it is three times thicker than the other steels, hence a decrease in the efficiency of the cooling device was observed. Nevertheless, the temperature reached by the UNS S32101 was not high enough to fall into the secondary phases’ temperature stability regime. An increase in temperature within the experimental error, with some differences due to the experimental set-up, was observed for the other three DSS grades.

### 3.3. Mechanical Behavior

**Figure 8:**
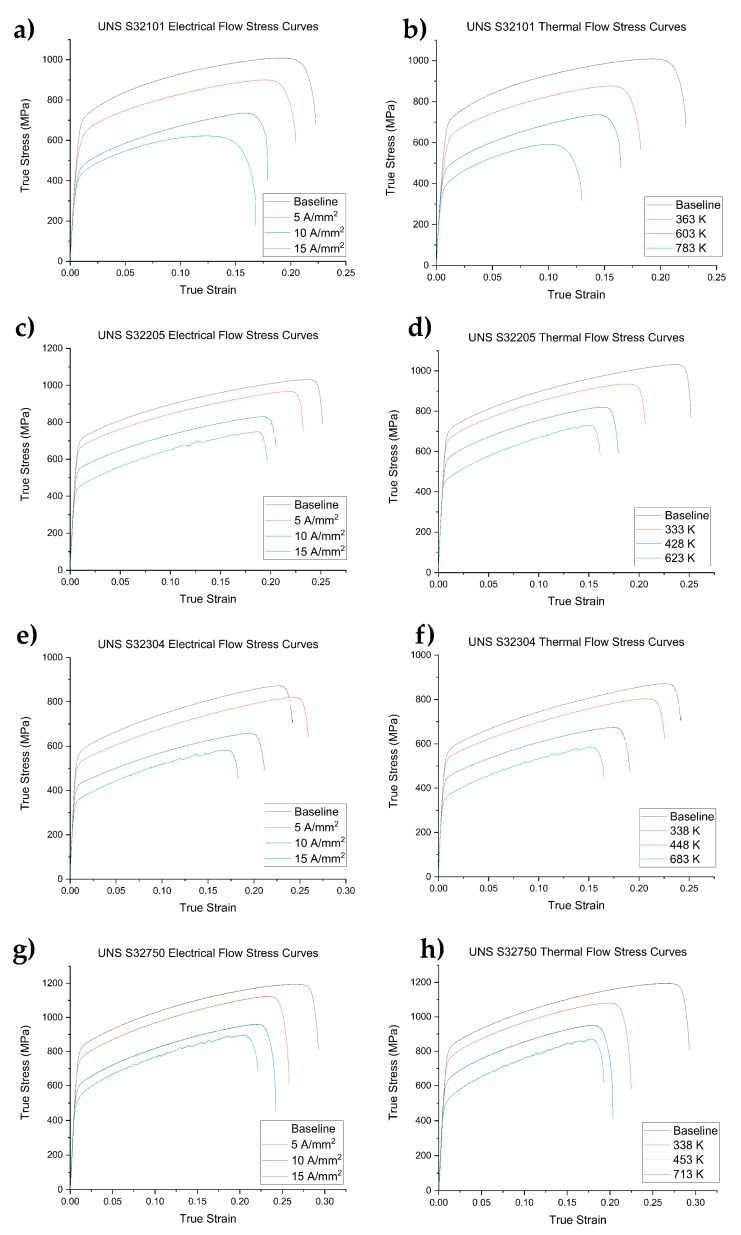
True stress-strain curves of electrical current tests on the left side and the related thermal counterpart on the right side for the different DSS grades: UNS S32101 (**a**,**b**); UNS S32205 (**c**,**d**); UNS S32304 (**e**,**f**); and UNS S32750 (**g**,**h**).

The flow stress curves obtained with the different current densities and the thermal counterparts of all tested materials are shown in Figure 8. The Room Temperature (RT) tensile test was considered as the baseline (black flow stress curves in Figure 8). The electrical tests, as well as the thermal counterparts, showed a reduction in the total elongation as the temperature and the current densities increased, except for the UNS S32304, which showed an increase in fracture strain at 5 A/mm^2^ compared to the base material (Figure 8e, red curve). Mechanical properties, such as yield stress and Ultimate Tensile Strength (UTS), showed the same trend as the total elongation, decreasing with increasing current density and temperature. A peculiar morphology of the flow stress curves for the tests conducted at 15 A/mm^2^ along with the thermal counterpart were noted for all DSS grades, except for UNS S32101. The segmentation of the flow stress curves was related to a phenomenon known as Dynamic Strain Aging (DSA) [59,60,61], which is an interaction between solid solution elements and the moving dislocations. UNS S32101, on the other hand, did not show DSA because the test temperature was high enough to facilitate the depinning of the dislocation from the solute atoms and because of the increased diffusion rate. The influence of electrical current on the DSA is beyond the scope of this paper. The highest homologous temperature reached by the UNS S32101 was 0.45 (Table 3), which is very close to the threshold that separates the cold/warm deformation regime from the hot one. This is why the shape of the flow stress curves (electrical and thermal) for higher temperature tests of UNS S32101 was different from the others DSS grades.

As mentioned above, total elongation is not a reliable parameter to consider when trying to understand the influence of electrical current on the plastic flow of metals in any type of deformation that involves a localized diminishing in the cross section during the test. To understand the influence of electrical current on these materials, the elongation corresponding to UTS, defined as uniform elongation, was used to compare the behavior of the different DSS grades according to the method in [62,63]. Up to this point, the absence of necking effects did not lead to any considerable reduction of the sample cross section and thus related temperature increases due to higher current density. However, after necking occurred, the stress distribution was no longer uniaxial and the stress triaxiality was not constant.

Relative uniform elongation was calculated for all the DSS grades with the following equation:

(3) $$\varepsilon_{UTS\;rel}=\frac{\varepsilon_{UTS\;i}}{\varepsilon_{UTS\;baseline}}$$

in which $\varepsilon_{UTS\;i}$ is the test uniform elongation and $\varepsilon_{UTS\;baseline}$ is the reference uniform elongation (i.e., uniform elongation of the RT tests).

**Figure 9:**
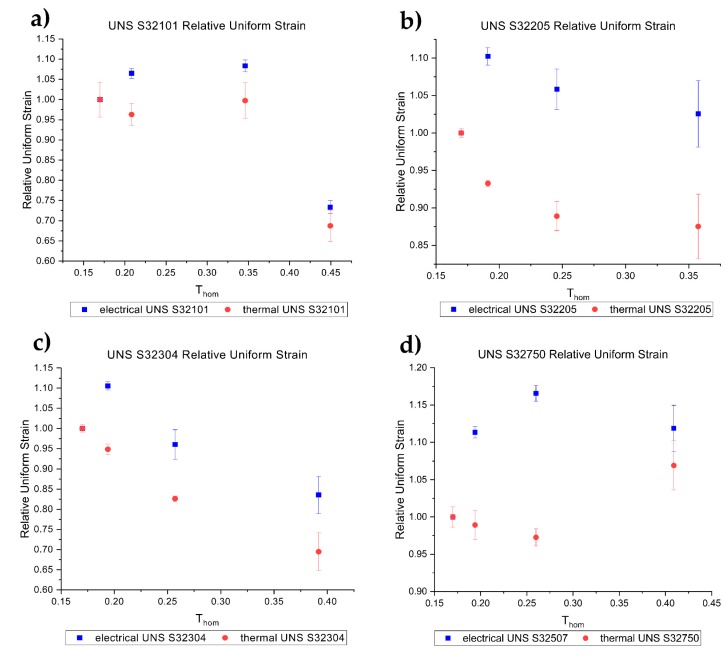
Relative uniform elongation of the different DSS grades. Red circles refer to the thermal test while the blue dots to the electrical test. UNS S32101 (**a**), UNS S32205 (**b**), UNS S32304 (**c**) and UNS S32750 (**d**).

Figure 9 shows the relative uniform elongation of the different DSS grades. The red circles refer to the thermal test while the blue dots to the electrical test showing the relative uniform elongation of the electrical and thermal tensile tests of the DSS grades tested. It can be seen that the uniform elongation of the electrical tests was higher compared to that of the thermal counterparts for all the DSS grades. There was not a significative correlation between relative uniform elongation and test temperature for the different grades. Moreover, an opposite trend was observed for the electrical current test compared to the thermal ones for UNS S32750 and UNS S32205.

A large uncertainty was calculated in relatively uniform elongation for both the thermal and the electrical tests for all DSS grades except for the UNS S32101, because of the difficulty in measuring the uniform elongation in the case of insurgence of DSA phenomenon. Furthermore, it is interesting to note the positive effect of the electrical current on the uniform elongation compared to the thermal counterpart. The electrical tensile test for almost all materials, except UNS S32304 at 10 A/mm^2^ and 15 A/mm^2^ as well as UNS S32101 at 15 A/mm^2^, showed a uniform elongation higher than the baseline in contraposition with the thermal counterpart, denoting an influence of the electrical current on the plastic flow. There was an evident correlation between the current density and the increase in relative uniform elongation. EPE appeared to have a smaller effect on the uniform elongation of UNS S32101 and UNS S32304 compared to the other grades, while the most prominent effect was observed for UNS S32750. UNS S32101 and UNS S32304 are known as lean duplex stainless steel because of the low amount of alloying elements compared to the standard DSS UNS S32205 and the high alloyed DSS UNS S32750. The major effect of the electrical current on the uniform elongation was observed for UNS S32750, which showed an increase of up to 17.5% compared to the baseline for the test conducted at 10 A/mm^2^. The different behavior of the two lean DSSs compared to the other grades is probably because of the different work hardening rate due to the different alloying elements. An in-depth study on the effect of electrical current on the work hardening rate is needed.

**Figure 10:**
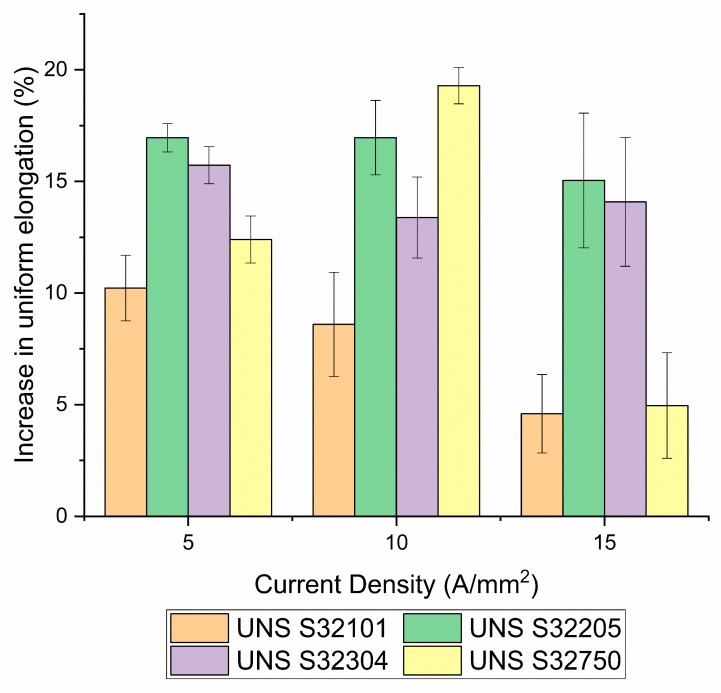
Uniform elongation percentage increase of electrical tests compared to the thermal ones with respect to current density.

Figure 10 depicts the increase in uniform elongation of the electrical tests compared to the thermal counterparts computed as the difference between the relatively uniform elongation of the electrical and the thermal tests. The highest increase in uniform elongation (19.3%) compared to the other DSSs was observed for the test conducted on UNS S32750 at 10 A/mm^2^. UNS S32205 and UNS S32304 reached almost the same increase in uniform elongation with respect to the current density while a gradual decrease was observed for UNS S32101. A particular trend within the tested materials was not observed. Nevertheless all DSS grades showed an increase in uniform elongation approximately between 5% and 20% in the current density range tested.

## 4. Discussion

In a previously published work, a SFE dependence of EPE on the total elongation has been observed [32]. High SFE materials show a better formability compared to the low SFE ones.

The microstructure of DSS consists of a high SFE phase (ferrite), in which a low SFE phase (austenite) nucleates and grows as the alloy cools. In the present study, a positive net effect of electrical current was observed regardless of the presence of a low SFE phase, which should decrease the total elongation. Zhao et al. [64] conducted some numerical simulations regarding the electrical potential inside a nanocrystalline material, taking into account non-homogeneities such as grain boundaries. They found that there could be a non-uniform distribution of electrical potential, which leads to an uneven distribution of electrical current inside the material. This means that, in regions with higher electrical resistivity, such as grain and phase boundaries and dislocation cell walls, a higher localized resistive heating can be presumed. The same hypothesis was made by Sànchez et al. [42] on an AISI 308L austenitic stainless steel subjected to electroplastic drawing and magnesium alloy AZ31 under uniaxial micro-tension. They observed a lower microhardness for the material subjected to electropulsing heat treatment after drawing in comparison with the conventional ones in the case of the AISI 308L and an increase in fracture strain of the AZ31. They concluded that a microscale hot spots should be present in both materials, reducing the hardness and changing the texture in the first case while arresting microcracks propagation and voids initiation in the latter.

Current distribution inside DSSs can be affected by the different electrical resistivity of ferrite and austenite due to their different crystal structure and alloying elements. In fact, ferrite matrix percolates the entire microstructure and has a lower electrical resistivity compared to austenite [65]; hence, an uneven distribution of the electrical current can be expected. The different work hardening rate of the two phases has to be taken into account as well: austenite is subjected to higher deformation, hence higher work hardening rate owing to its low SFE compared to ferrite is expected [66]. Since ferrite has a higher yield stress than austenite, most of the plastic deformation initiates in the austenitic grains, increasing the dislocation density of that phase, which affects the resistivity as well. Inhomogeneities in the microstructure such as dislocations network, secondary phases precipitates, phase and grain boundaries could cause a stagnation of electrons in their proximity, as stated by Ruszkiewicz et al. and observed by Zhao et al. [33,64]. The local increase of electrons changes the electron to atom ratio, which can lead to a decrease in the bond strength of the material, easing the plastic deformation. The uneven distribution of electrical current through the microstructure and inside the grains can also lead to a localized resistive heating favoring the development of texture due to crystal rotation, as observed by Rahnama et al. and Sànchez et al. [42,45], and can also aid the diffusion rate because of an increase in atom flux due to the electrical current [67], a phenomenon known as electromigration. Even though some researchers claimed that the athermal effect (i.e., electron wind force) plays an insignificant role in EPE [28,29], the increase in current density in the grain and phase boundaries can increase the effect of the electron wind force, aiding the plastic flow of the material, as confirmed by the lowering of the electrical resistivity.

The different effect of EPE on the DSSs is probably because of the different work hardening rate due to the different composition, as stated before, and also because of the different grain size distribution of the phase inside the material, which can affect the amount of phase and grain boundaries.

To summarize, the increase in uniform elongation with respect to the thermal tests in DSSs could be related to the aforementioned phenomena. In particular, we suggest that the uneven distribution of the electrical current throughout the microstructure plays a significant role in aiding the plastic flow, regardless of the presence of low SFE austenite, which has been shown to reach fracture prematurely when deformed under applied continuous electrical current [32,40].

## 5. Conclusions

Tensile tests with the aid of electrical current and corresponding thermal tests were conducted on four DSS grades to investigate the influence of electrical current on materials that possess very different SFE phases. Comparisons between the mechanical properties of electrical tests and the thermal counterparts were performed.

Thermal regimes reached by the DSS grades are within the cold/warm range and are comparable to each other except for UNS S32101 because of its lower surface to volume ratio due its greater thickness compared to the other DSSs.

No differences in terms of yield stress and ultimate tensile strength were found between the electrical and the thermal tests. On the other hand, a clear effect of the electrical current on the uniform elongation and on the total elongation was observed.

All tested materials showed an increase in uniform elongation compared to the thermal tests and to the baseline as well as the total elongation.

Standard DSS UNS S32205 and Super DSS UNS S32750 showed the biggest increase in uniform elongation for the electrical tests compared to the thermal tests, the baseline and to the other DSS grades. The highest increase in uniform elongation was approximately 20% for UNS S32750 at 10 A/mm^2^. Nevertheless, all tested materials showed an increase in uniform elongation between 5% and 20% compared to the thermal tests. The lowest was UNS S32101 at 15 A/mm^2^ because of its higher thermal regime due to specimen geometry.

This novel manufacturing process for this type of stainless steels could be useful in industrial application such as wire drawing to substitute the external armor of submarine communication cables, in order to prevent seawater corrosion in the case that the outside polymeric insulation is damaged.

## Figures and Tables

**Table 1:** Chemical composition of the studied materials (%wt).

	C	Si	Mn	Cr	Ni	Mo	N	P	S	Cu	Ti
UNS S32101	0.025	0.65	5.13	21.57	1.56	0.28	0.229	0.019	0.001	0.3	-
UNS S32304	0.03	0.56	1.43	23.17	4.29	0.18	0.13	0.027	0.001	0.16	-
UNS S32205	0.027	0.58	1.52	22.75	5.04	3.19	0.16	0.027	0.001	-	-
UNS S32705	0.014	0.35	0.68	24.99	3.63	6.41	0.253	0.021	0.001	0.06	0.002

**Table 2:** Austenite and ferrite volume fraction of the different Duplex Stainless Steel grades.

DSS Grade	Austenite	Ferrite
UNS S32101	0.51 ± 0.02	0.49 ± 0.03
UNS S32205	0.53 ± 0.03	0.47 ± 0.04
UNS S32304	0.51 ± 0.01	0.49 ± 0.02
UNS S32750	0.48 ± 0.04	0.52 ± 0.05

**Table 3:** Current density, temperature and homologous temperature of the investigated DSSs.

DSS Grade	Current Density (A/mm^2^)	Mean Temperature (K)	Homologous Temperature (ad)
UNS S32101	5	363	0.21
	10	603	0.35
	15	783	0.45
UNS S32205	5	333	0.19
	10	428	0.25
	15	623	0.36
UNS S32304	5	338	0.19
	10	448	0.26
	15	683	0.39
UNS S32750	5	338	0.19
	10	453	0.26
	15	713	0.41
